# Supplementary material for: Ion exchange chromatography as a simple and scalable method to isolate biologically active small extracellular vesicles from conditioned media
Source: PLoS One. 2023 Sep 15;18(9):e0291589. doi: 10.1371/journal.pone.0291589 (PMC10503763; doi:10.1371/journal.pone.0291589)
Supplement: S1 File — Step-by-step protocol, also available on protocols.io. (PDF) [file pone.0291589.s001.pdf]

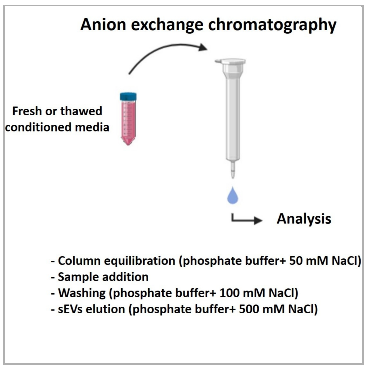

Ion exchange chromatography for small extracellular vesicles isolation

Ricardo Malvicini[1,2,3,4], Diego Santa Cruz[1], Anna Maria Tolomeo[4,5], Maurizio Muraca[2,3,4], Gustavo Yannarelli[1], Natalia Pacienza[1]

[1]Instituto de Medicina traslacional, trasplante y bioingeniería (IMETTyB)-CONICET;
[2]Dipartimento della salute della donna e del bambino, Università di Padova;
[3]Laboratory of Extracellular Vesicles as Therapeutic Tools, Fondazione Istituto di Ricerca Pediatrica Città della Speranza, Padua, Italy;
[4]L.i.f.e.L.a.b. Program, Consorzio per la Ricerca Sanitaria (CORIS), Padua, Italy;
[5]Department of Cardiac, Thoracic and Vascular Science and Public Health, University of Padova, Padua, Italy

Ricardo Malvicini

Protocol Info: Ricardo Malvicini, Diego Santa Cruz, Anna Maria Tolomeo, Maurizio Muraca, Gustavo Yannarelli, Natalia Pacienza . Ion exchange chromatography for small extracellular vesicles isolation. **protocols.io** https://protocols.io/view/ion-exchange-chromatography-for-small-extracellula-b3qrqmv6

Created: Jan 12, 2022

Last Modified: Jun 06, 2023

PROTOCOL integer ID: 56817

Keywords: Ion Exchange Chromatography; Extracellular Vesicles; Isolation method; conditioned media

ABSTRACT

In the last few years, extracellular vesicles have become of great interest due to its potential as biomarkers, drug delivery systems and, in particular, as therapeutic agents. However, there is no consensus on which is the best way to isolate these vesicles. The choice of the isolation method depends on the starting material (i.e conditioned culture media, urine, serum, ecc) and their downstream applications. Even though there are numerous methods to isolate sEV, few of them are compatible with clinical applications, as they are not scalable. In the present work, we set up a protocol to isolate sEV from conditioned culture media by ion exchange chromatography, which is a simple, fast and scalable method, suitable for clinical production of sEV. We performed the isolation using an anion exchange resin (Q sepharose) and eluted the sEV using 500mM NaCl. We characterized the elution profile by measuring protein and lipid concentration and CD63 by ELISA. Moreover, we immunophenotyped all the eluted fractions, evaluated the presence of TSG101, calnexin and cytochrome C by western blot and analysed nanoparticle size and distribution by NTA and tRPS and morphology by TEM. Finally, we evaluated the immunomodulatory activity *in vitro*. We found that most sEV are eluted and concentrated in fraction 4, with a mean size <150nm, which are positive for CD9, CD63, CD81 and TSG101, while most proteins are eluted in fraction 5. Moreover, sEV in fraction 4 exerted an anti-inflammatory activity on LPS-stimulated macrophages. In summary, we set up a scalable and clinically compatible method to chromatographically isolate small extracellular vesicles, from conditioned culture media, that retain their biological activity.

GUIDELINES

- An Ion Exchange Chromatography method to isolate mesenchymal stromal cells-derived extracellular vesicles from conditioned media.

MATERIALS

Materials& Reagents:

(Equilibrate all materials to room temperature before experiment)

1. Microtubes (1.5mL):

Input	Flow through	Wash	No.1	No.2	No.3	No.4	No.5	No.6	No.7	No.8

2. Chromatography Columns (Bio-Rad, 7321010)
3. Poly Column Rack (Bio-Rad, 7317005) (optional)
4. Anion exchange resin (Q Sepharose Fast Flow, GE Health Care Life Science, CAT# 17051001)
5. Equilibration Buffer: 50mM phosphate buffer, **50mM** NaCl pH=7.5

1M phosphate buffer	5M NaCl	sterile distilled water	Total Volume
25mL	5mL	470mL	500mL

6. Wash Buffer: 50mM phosphate buffer, **100mM** NaCl pH=7.5

1M phosphate buffer	5M NaCl	sterile distilled water	Total Volume
25mL	10mL	465mL	500mL

7. Elution Buffer①: 50mM phosphate buffer, **500 mM** NaCl pH=7.5

1M phosphate buffer	5M NaCl	sterile distilled water	Total Volume
25mL	50mL	425mL	500mL

8. NaOH 1M (Sigma, CAT# 137031)

9.Acetic acid 1M (Sigma, CAT#1.60305)

Notes for Buffer 5-7:

a. 1M phosphate buffer solution (Sigma, CAT# S9888-500G); NaCl (Sigma, CAT# S9888-500G)

b. The buffers should be filtered with 0.22μm strainer (Thermo, 194-2520) to keep sterile.

SAFETY WARNINGS

No safety warnings

BEFORE START INSTRUCTIONS

After collecting the conditioned media, centrifuge it at 2500xg for 10 minutes in order to eliminate cell debris. After that, filter it with a 0.22µM filter, in order to eliminate the microvesicles. At this point, you can either freeze the medium or process it immediately. It is advisable to process it right away to avoid freezing-thaw cycles.
For the chromatography, equilibrate all materials and buffers to room temperature.

Ion Exchange Chromatography

2h 50m

1 Mix well and add 5 mL of anion exchange resin (Q Sepharose Fast Flow, GE Health Care Life Science, CAT# 17051001) to an empty column (Bio-Rad, 7321010) at RT and wait for the resin to sediment; remove the end cap and let the ethanol flow out. Connect the end cap, 4 mL of resin bed volume should remain in the column; 5m

2 Gradually add at least 10 column volumes (CV) (4 mL*10=40 mL) of Equilibration Buffer (Note: do it gently to prevent making bubbles. Cutting off the end of a 1000 µL tip and placing it against the column wall can help loading the buffer into the column). Let the buffer flow out slowly. Leave at least 10 mL of Equilibration Buffer in the column, connect the end cap for stopping eluent flow, and let the resin to pack overnight (Note: a well-packed column will contribute significantly to resolution); 30m

3 The day after, process the conditioned media. If it was frozen, thaw it at 37°C, otherwise process it immediately. Collect 1 mL of the conditioned medium into the tube (input fraction). Then, add the previously filtered media to the column. Do it softly to prevent disrupting the surface of the resin (Note: cutting off the end of a 1000 µL tip and placing it against the column wall can help loading the media into the column) and let the media flow out slowly. Collect 1 mL flow-through media into the tube in the last stage of sample loading; 1h 30m

4 Once all the conditioned media has flown through, gradually add 10 CV (4 mL*10=40 mL) of Wash Buffer and let the buffer flow out slowly. Collect 1 mL Wash buffer into the tube in the middle stage of washing; 30m

5 Connect the end cap, add 2 CV (4 mL*2=8 mL) of Elution Buffer to the column, remove the end cap, and collect the eluates. Collect 8 fractions of 1 mL each. This way, there is less risk of disturbing the resin bed surface. 15m
Otherwise, add 1 mL of Elution buffer to the column each time and collect the flow-out buffer as a fraction. Repeat eight times to collect 8 fractions in 8 tubes (No.1 to No.8) with a total volume about 8 mL. Usually, Fraction 4 contains most of the EVs.

6 Finally, take aliquots from each fraction to assess particle number, protein content, lipid content, and other downstream analysis. Depending on the downstream applications, it may be necessary to desalt the samples, as they are eluted with a 500 mM NaCl buffer (Note: it can be easily done with 100KDa Amicon filters). It is highly recommended that fraction 4 is further aliquoted, to avoid freeze-thaw cycles. All the samples should be stored at -80°C. (Note: assessing protein concentration in the input, flow-through, and wash fractions along with the

eluted fractions, is useful to calculate the concentration factor).

Resin regeneration (optional)

4h 15m

7 Once all the fractions have been collected, wash the column with 10 CV (4 mL*10=40 ml) of 2.0M NaCl (contact time => 15 min). 15m

8 Then, wash the column with 10 CV (4 mL*10=40 ml) of 1.0M NaOH (contact time = 2 h). 2h

9 Finally, wash the column with 10 CV (4 mL*10=40 ml) of 1.0M Acetic acid (contact time = 2 h). 2h

10 Store the equilibrated resin in ethanol at +4°C (for a volume of 4 mL of resin add 1mL of ethanol) or equilibrate the column with 10 CV (4 mL*10=40 ml) of Equilibration buffer to use it.
