# Supplementary material for: Ion exchange chromatography as a simple and scalable method to isolate biologically active small extracellular vesicles from conditioned media
Source: PLoS One. 2023 Sep 15;18(9):e0291589. doi: 10.1371/journal.pone.0291589 (PMC10503763; doi:10.1371/journal.pone.0291589)
Supplement: S2 File — (DOCX) [file pone.0291589.s002.docx]

**Supporting information file 2 (S2)**

**MSCs culture**

Human umbilical cord perivascular cells (HUCPVCs) derived mesenchymal stromal cells were MSCs were bought from Promocell. HUCPVCs were cultured as previously described [1], expanded until passage 4, and characterized according to the International Society for Cellular Therapy (ISCT) guidelines [2]. Briefly, passage 3-4 HUCPVCs were thawed and cultured in Dulbecco’s modified Eagle’s medium low-glucose (DMEM, Gibco) supplemented with 10% of fetal bovine serum (FBS, Gibco), 100 U/ml penicillin, and 100 μg/ml streptomycin (Gibco) at 37°C in a humidified incubator containing 5% CO_2_. Then, cells were collected using Trypsin 0.1% in Hank’s Buffer (Gibco) and plated in 175 cm^2^ culture flasks at a density of 4000 cell/cm^2^ until 70% confluence. Finally, culture media was changed to minimum essential Eagle medium alpha without phenol red (α-MEM, Gibco) nor FBS for 48 h. After that, conditioned media was collected and centrifuged at 2500 ×g for 10 min to eliminate cellular debris and then filtered by 0.22µM (filter unit syringe driven, Millex-GP). We performed 5 independent isolations and collected between 60-200mL of conditioned media, to evaluate the robustness and the reproducibility of the isolation protocol. The conditioned media collected contained the sEV produced by approximately 9-30x10^6^ MSC or 3-10 T175 cm^2^ flasks. The supernatant was either processed immediately or stored at -80°C until further processing.

**Tunable resistive pulse sensing (tRPS)**

Particle concentration and size distribution were analysed by Tunable Resistive Pulse Sensing (TRPS) technology with the qNano instrument (Izon Science, Christchurch, New Zealand). For the analysis, a NP100 or NP150 membrane was used. The concentration of particles was standardized using a CPC100 calibration solution diluted 1:10000 (110 nm mean carboxylate polystyrene beads; raw concentration 1.00E+12) [3].

**Transmission electron microscopy**

One drop (5 µL) of each fraction was placed on 400 mesh holey film grid; after staining with 1% uranyl acetate for 2 min the sample was washed with PBS and then observed with a Tecnai G2 (FEI) transmission electron microscope operating at 100 kV. Images were captured with a Veleta (Olympus Soft Imaging System) digital camera [1].

**Immunophenotyping of MSC-sEVs**

The immunophenotyping of the diverse fractions was performed by flow cytometry using the MacsPlex Exosome Kit (Miltenyi Biotec) following manufacturer’s instructions. Briefly, 1x10^9^ particles were loaded in a 1.5 ml tube and diluted to 120µl with MACSPlex buffer (in the samples in which no particles were detected, a volume equal to the maximum volume loaded was used). Then, 15 µl exosome capture beads and 15 µl of antibody mixed were added to each tube and incubated for 1h at room temperature in agitation. Then, 500 µl of MACSPlex buffer was added to each tube and they were centrifuged at 3000g for 5 min. Then, samples were washed with 500 µl of MACSPlex buffer and centrifuged again. Finally, 500 µl of the supernatant were discarded, the samples were resuspended and transferred to a flow cytometry tube. Data was collected and analysed using a Cytoflex (Beckman Coulter) flow cytometer.  For blank control, only MACSPlex buffer was used.

**Protein quantification**

Protein concentration was measured using the Pierce™ BCA Protein Assay Kit (ThermoFisher Scientific) according to manufacturer’s instructions. Briefly, 25 µl of each fraction was loaded in a 96 well plate, mixed with 200 µl of BCA reagent and incubated for 30 min at 37°C. Absorbance was read at 562nm.

**Lipid quantification**

Lipid content was assessed using the sulpho-phospho-vanillin assay [4]. Briefly, 70 µl of each sample was mixed with 200 µl of 96% sulphuric acid and incubated 20 min at 90°C. Once cooled down, 120 μl of 1 mg/ml phospho-vanillin reagent in 17% H_3_PO_4_ was added, vortexed, and incubated for 1 h at room temperature. Finally, absorbance was measured at 540nm.

**ELISA for CD63**

The presence of CD63 in the samples was assessed by a sandwich ELISA that specifically detects CD63 associated with vesicles [1]. Briefly, a 96 well plate (Nunc Maxisorp, ThermoFisher Scientific) was coated with 100 µl of CD63 antibody (Clone H5C6, Cat 556019, BD Pharmigen) and incubated overnight. Then, the plate was washed with PBS with 0.05% Tween-20 and blocked with BSA 1% in PBS. After washing, 50 µl MSC-sEV samples were incubated for 3 h at room temperature. After washing, a detection antibody (Cat 353017, Biolegend) was added and incubated for 1h at room temperature. After washing, the samples were incubated with Streptavidin-HRP (DY997, R&D Systems) for 20 min. After washing, TMB was added and incubated for 5 min. Finally, the reaction was stopped with 2 N sulfuric acid and absorbance was read at 450 nm.

**Western Blot**

For the western blot analysis, MSCs were lysed in RIPA buffer and both MSC and MSC-sEVs were incubated with Laemmli buffer with β-mercaptoethanol for 5 min at 95°C, for complete protein denaturation. Then, 5 µg of protein were loaded and resolved in an SDS-polyacrylamide 4-12% gel at 150V and blotted using semi-dry transfer for 7 min at 25V to polyvinylidene difluoride membranes (PVDF) (GE Healthcare Life science). Membranes were blocked with 5% BSA in TBS-Tween for 1 h at room temperature and then incubated with primary antibodies overnight (anti-TSG-101 antibody 1:1000 abcam, anti-calnexin antibody 1:1000 and anti-cytochrome C antibody 1:1000, Santa cruz biotechnology) diluted in 1% BSA in TBS-Tween. After washing, membranes were incubated with secondary antibodies (goat-anti rabbit-HRP 1:5000, 65-6120, Thermofisher and goat anti mouse-HRP 1:5000, abcam) for 1 h at room temperature. After washing, bands were evidenced by means of ECL plus Western blotting analysis system (32134, Thermofisher). The Precision Plus Protein Dual Color Standards (#1610374) was used as a molecular weight marker.

**SDS- Page and silver staining**

Fifteen µl of each fraction were loaded and resolved in an SDS-polyacrylamide 4-10% gel at 150V. For the silver staining, gels were washed in ultra-pure water and fixed for 1 h with a solution containing 50% methanol and 10% acetic acid. After that, gels were washed 3 times with 50% methanol and 3 times with ultrapure water and sensitized with 0.02% sodium thiosulphate for 1 min. Then, gels were stained with 0.2% silver nitrate for 20 min and rinsed with ultra-pure water. Finally, gels were developed with a solution containing 6% Na_2_CO_3_ and 0.0004% sodium thiosulphate until desired intensity was achieved. Finally, gels were washed with ultrapure water and incubated with a solution containing 50% methanol and 10% acetic acid for 10 min.

**In vitro macrophage assay**

We tested *in vitro* the biological activity of the different fractions on LPS-stimulated RAW 264.7 cells, as previously described [1]. Briefly, RAW cells were stimulated with 10 ng/mL of LPS, with or without dexamethasone (1 µg/mL) or 30 µL of each fraction. After 16 h, nitrite concentration was measured in the culture media by Griess reaction, by measuring the absorbance at 540 nm. The anti-inflammatory activity was calculated as the percentage of inhibition in nitrite production, with respect to the LPS alone.

**Statistical Analysis**

Continuous variables were expressed as mean ± standard deviation (SD). One-way analysis of variance (ANOVA) with the post hoc Tukey’s test was used for all the intergroup comparisons. The analysis was performed using Prism 8 (GraphPad Software, San Diego, CA, USA). A p-value < 0.05 was considered statistically significant. *P* values: **p*<0.05; ^#^*p*<0.01; ‡*p*<0.001.

1. Malvicini R, Santa-Cruz D, De Lazzari G, Tolomeo AM, Sanmartin C, Muraca M, et al. Macrophage bioassay standardization to assess the anti-inflammatory activity of mesenchymal stromal cell-derived small extracellular vesicles. Cytotherapy. 2022;24. doi:10.1016/J.JCYT.2022.05.011

2. Dominici M, Le Blanc K, Mueller I, Slaper-Cortenbach I, Marini FC, Krause DS, et al. Minimal criteria for defining multipotent mesenchymal stromal cells. The International Society for Cellular Therapy position statement. Cytotherapy. 2006;8: 315–317. doi:10.1080/14653240600855905

3. Tolomeo AM, Castagliuolo I, Piccoli M, Grassi M, Magarotto F, De Lazzari G, et al. Extracellular Vesicles Secreted by Mesenchymal Stromal Cells Exert Opposite Effects to Their Cells of Origin in Murine Sodium Dextran Sulfate-Induced Colitis. Front Immunol. 2021;12. doi:10.3389/FIMMU.2021.627605/FULL

4. Visnovitz T, Osteikoetxea X, Sódar BW, Mihály J, Lőrincz P, Vukman K V., et al. An improved 96 well plate format lipid quantification assay for standardisation of experiments with extracellular vesicles. J Extracell Vesicles. 2019;8. doi:10.1080/20013078.2019.1565263
