# Supplementary material for: Ion exchange chromatography as a simple and scalable method to isolate biologically active small extracellular vesicles from conditioned media
Source: PLoS One. 2023 Sep 15;18(9):e0291589. doi: 10.1371/journal.pone.0291589 (PMC10503763; doi:10.1371/journal.pone.0291589)
Supplement: S3 File — Uncropped and unadjusted images of the blots for TSG101, calnexin and Cytochrome C and the gels after silver-staining are provided. (PDF) [file pone.0291589.s003.pdf]

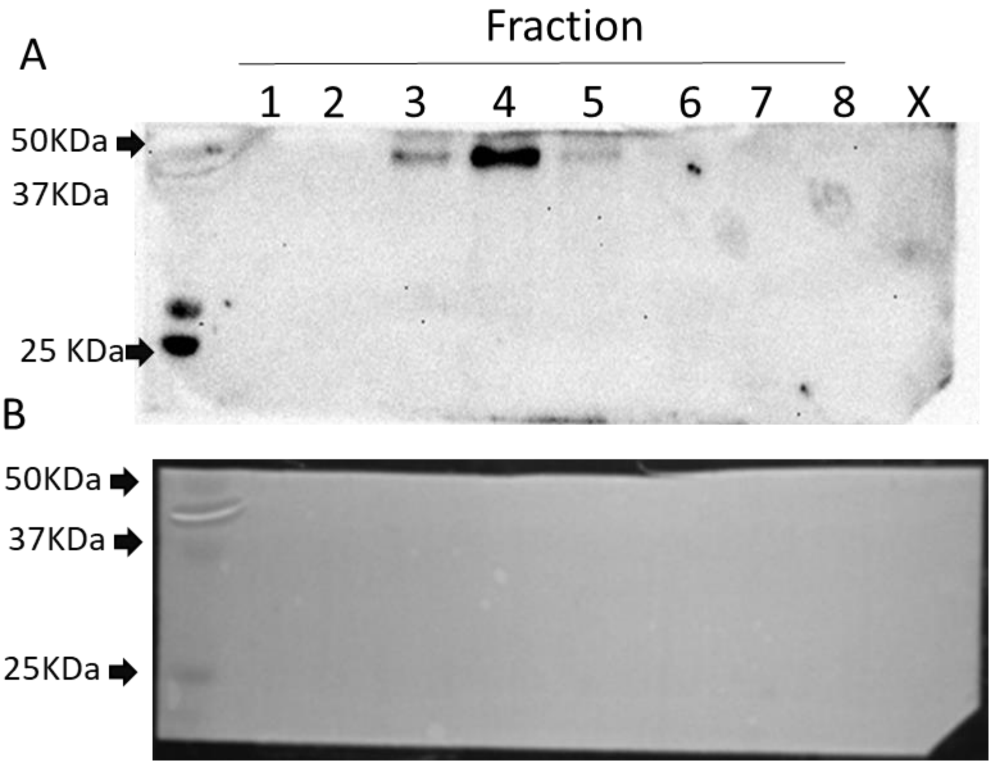

Blotting for TSG101- raw blot 1. Full membrane blot for TSG101 (A). The molecular weight markers can be observed at the left. Then, the different eluted fractions were loaded. The final lane was empty (X). Picture of the membrane before blotting (B). Pictures were taken using an IBright instrument (Invitrogen). This blot was used in Figure 5B.

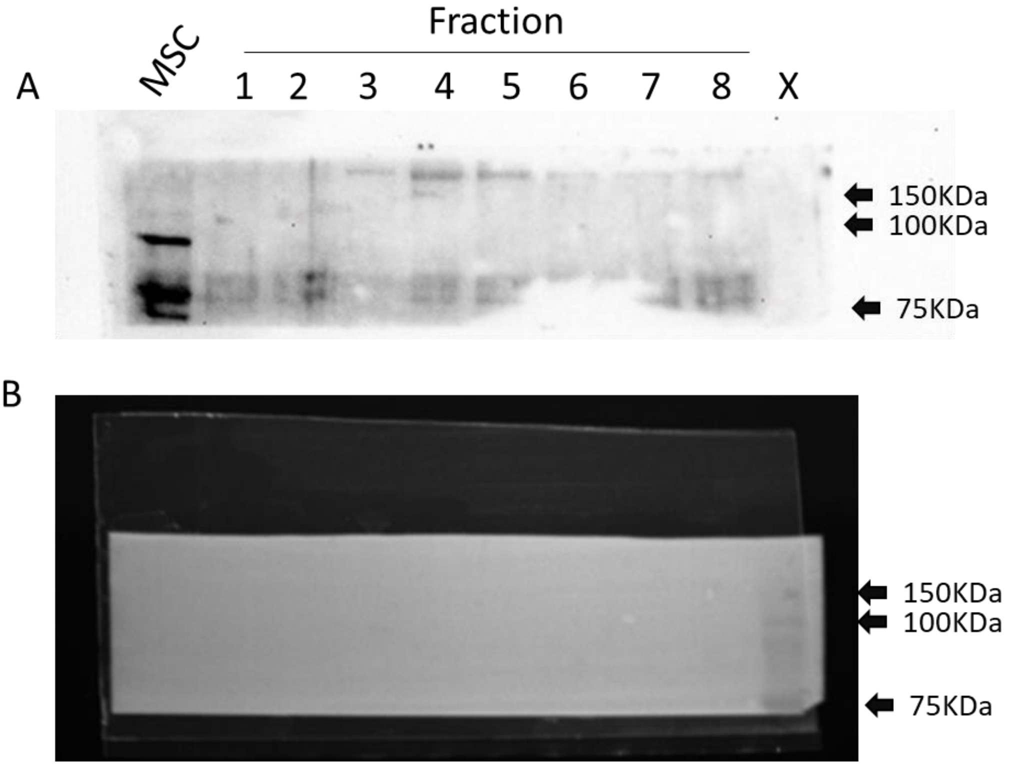

Blotting for Calnexin- raw blot 2. Full membrane blot for Calnexin (A). The molecular weight markers can be observed at the right. The MSCs were loaded as a positive control and different eluted fractions were also loaded. Picture of the membrane before blotting (B). Pictures were taken using an IBright instrument (Invitrogen). This blot was used in Figure 5C.

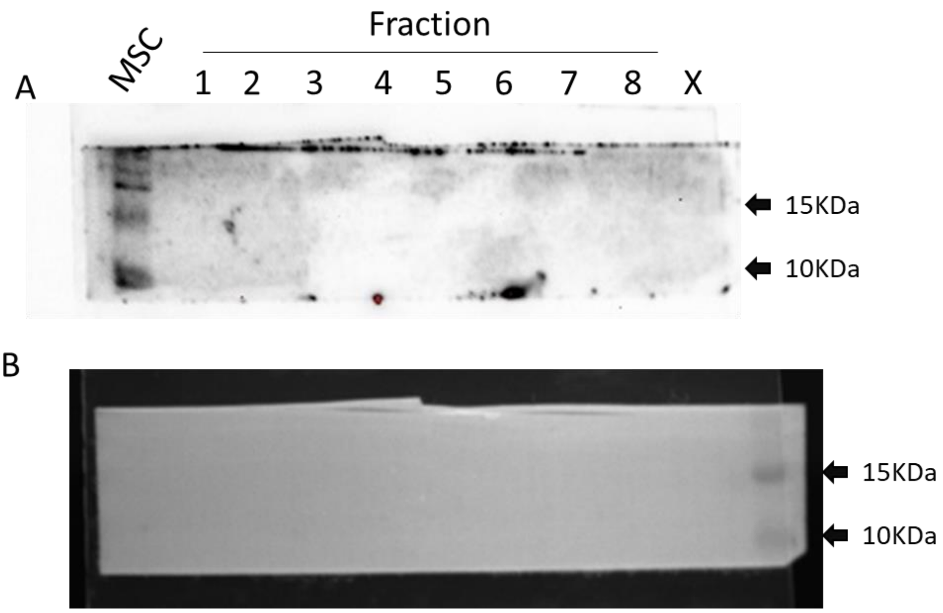

Blotting for Cytochrome C- raw blot 3. Full membrane blot for Cytochrome C (A). The molecular weight markers can be observed at the right. The MSCs were loaded as a positive control and different eluted fractions were also loaded. Picture of the membrane before blotting (B). Pictures were taken using an IBright instrument (Invitrogen). This blot was used in Figure 5C.

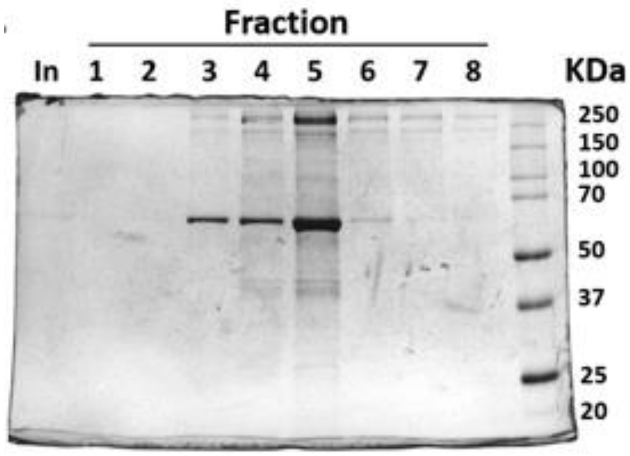

Full image of a gel after Silver staining. The input sample and all the eluted samples were loaded on a gel. The molecular weight marker can be seen on the right. Pictures were taken using an IBright instrument (Invitrogen). This picture was used in Figure 4B.

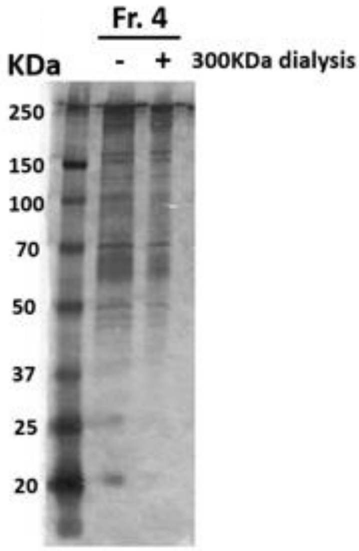

Full image of a gel after Silver staining. Fraction 4, where EVs are present, was dialyzed or not and samples were loaded on a gel and subjected to an SDS-Page, followed by silver staining. The molecular weight marker can be seen on the left. Pictures were taken using an IBright instrument (Invitrogen). This picture was used in Figure 4B.
