# Supplementary material for: Ion exchange chromatography as a simple and scalable method to isolate biologically active small extracellular vesicles from conditioned media
Source: PLoS One. 2023 Sep 15;18(9):e0291589. doi: 10.1371/journal.pone.0291589 (PMC10503763; doi:10.1371/journal.pone.0291589)
Supplement: S4 File — A representative gating strategy for the analysis of the different sample is provided. (PDF) [file pone.0291589.s004.pdf]

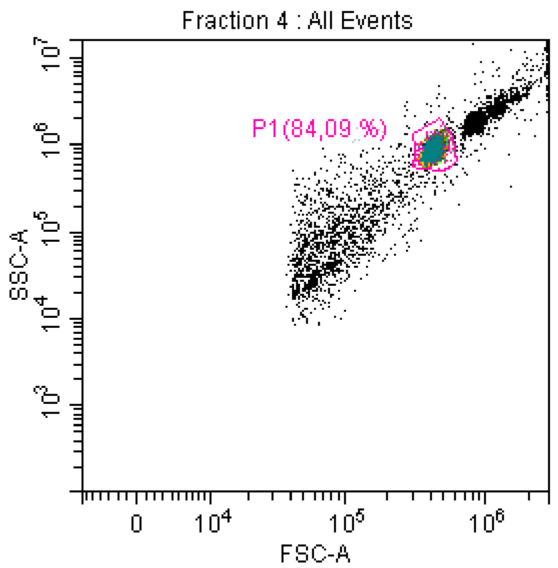

Representative gating strategy for the MacsPlex analysis and the EV surface proteins assessment 1. FSC-A vs SSC-A to select single beads population.

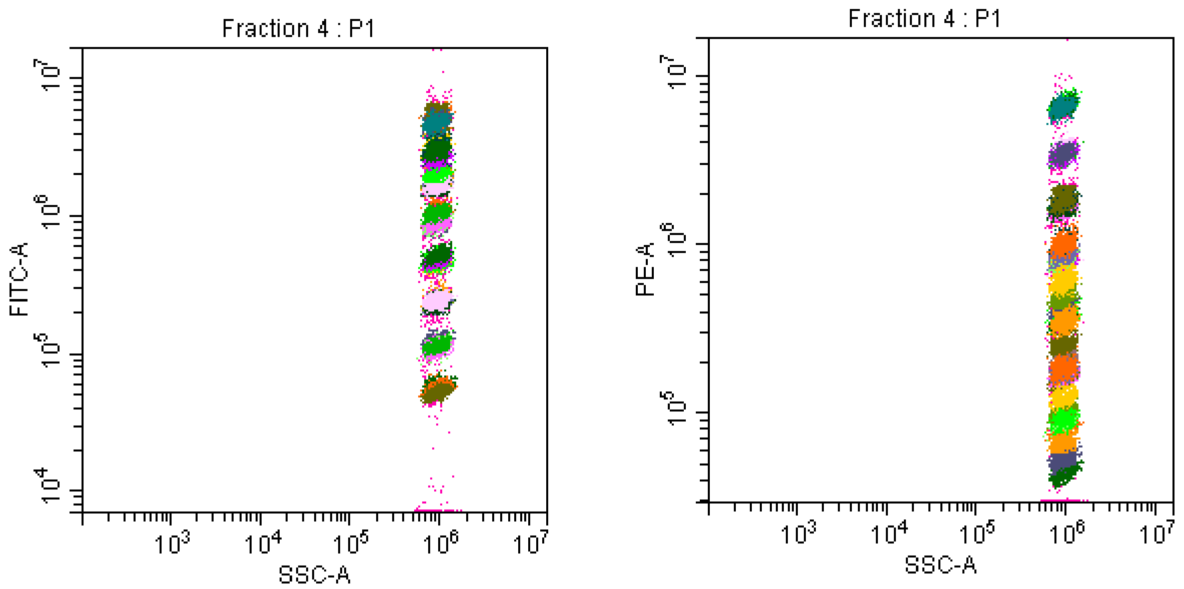

Representative gating strategy for the MacsPlex analysis and the EV surface proteins assessment 2. Evaluation of the different fluorescence of the different beads populations in FITC-A and PE-A

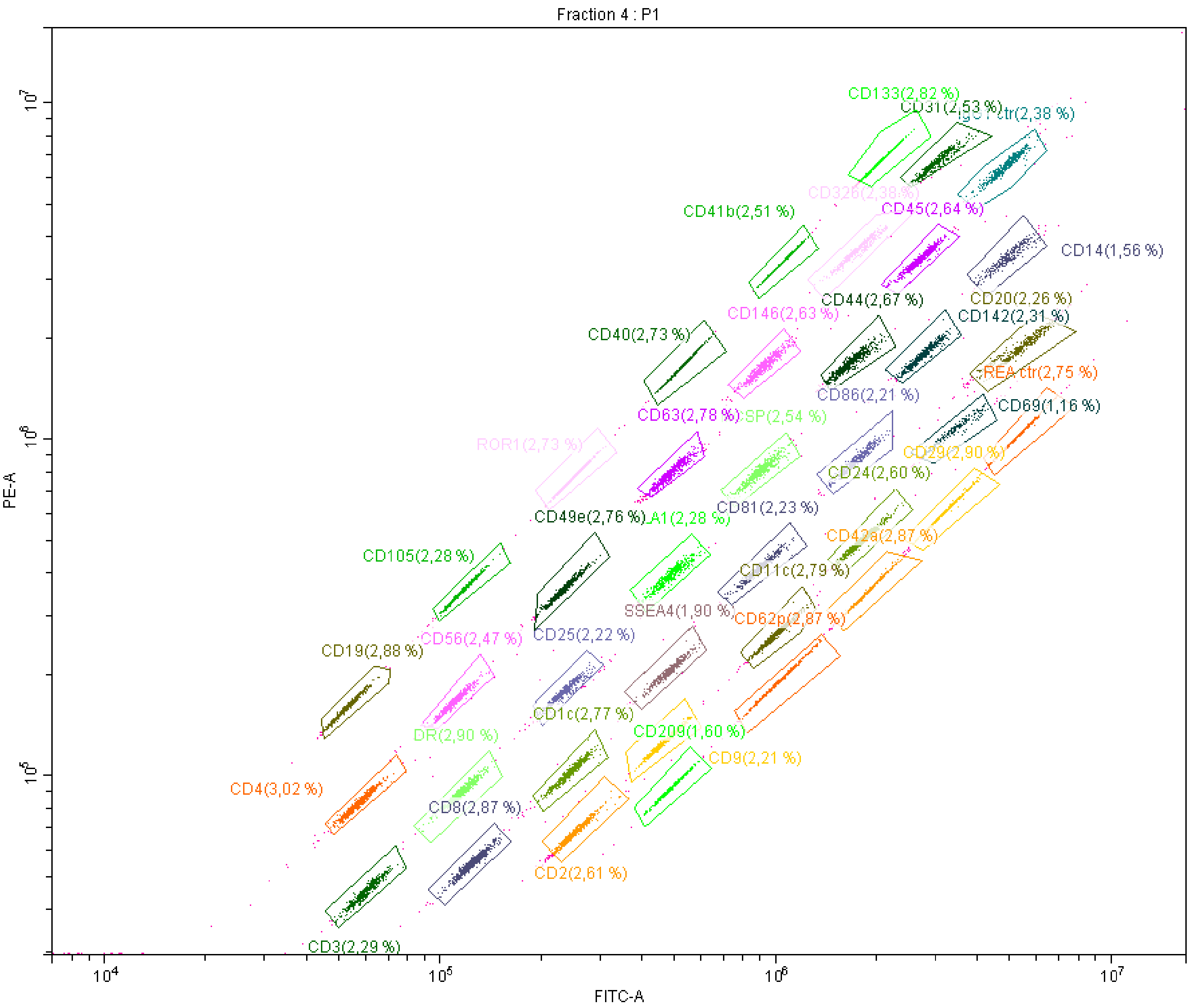

Representative gating strategy for the MacsPlex analysis and the EV surface proteins assessment 3. Evaluation of the single bead population for each surface marker. Each population was identified following the manufacturers'' instructions.
