# Supplementary material for: Ion exchange chromatography as a simple and scalable method to isolate biologically active small extracellular vesicles from conditioned media
Source: PLoS One. 2023 Sep 15;18(9):e0291589. doi: 10.1371/journal.pone.0291589 (PMC10503763; doi:10.1371/journal.pone.0291589)
Supplement: S1 Fig — The presence of surface proteins was assessed by flow cytometry using the MACSPlex kit. MSC related proteins are depicted in (A), adhesion molecules are shown in (B) and immunological related proteins are shown in (C). (DOCX) [file pone.0291589.s006.docx]

**Supplementary material**

**
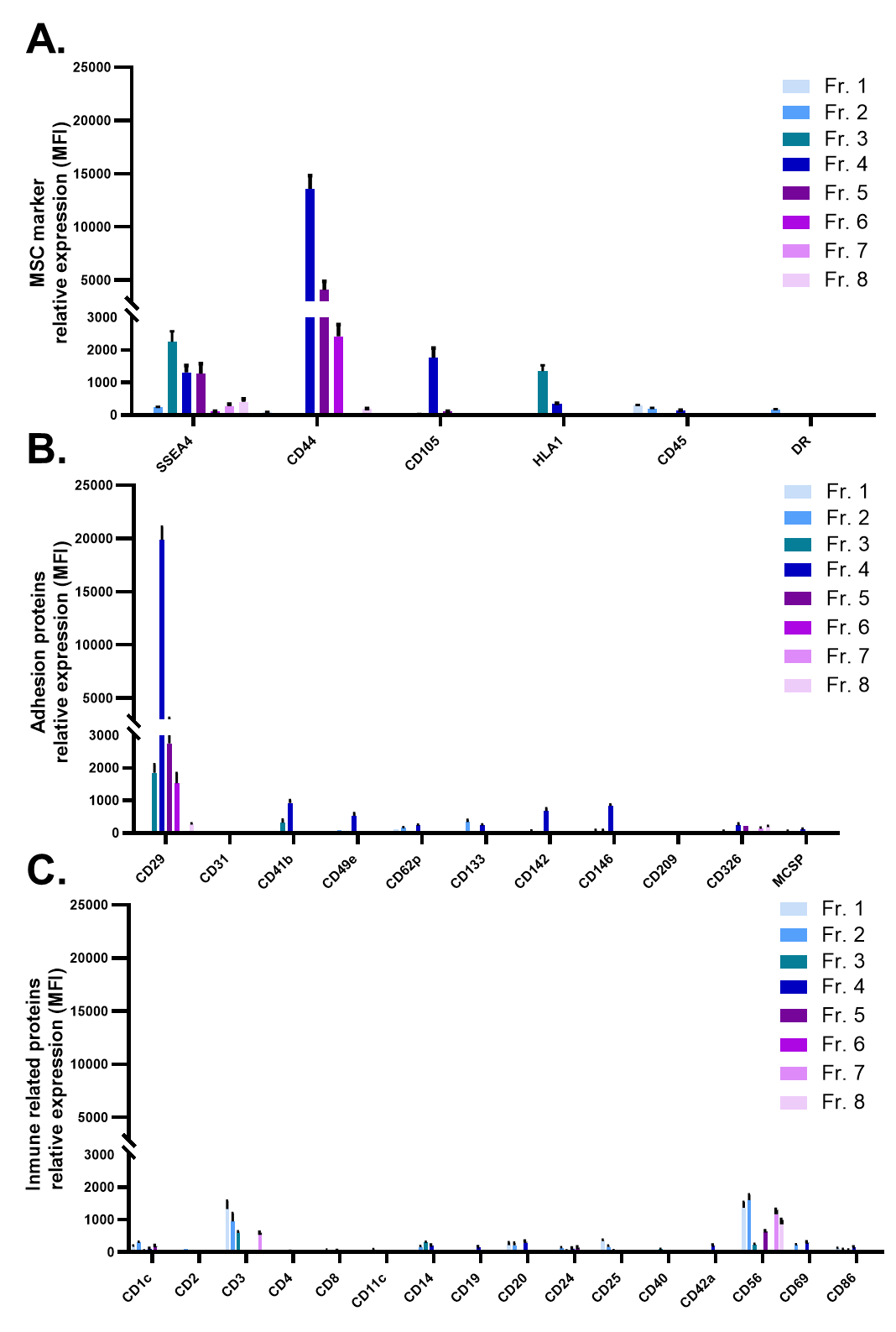
Supplementary Figure 1**

**Fig S1. sEV surface proteins assessment.** The presence of surface proteins was assessed by flow cytometry using the MACSPlex kit. MSC related proteins are depicted in **(A),** adhesion molecules are shown in **(B)** and immunological related proteins are shown in **(C).**
