# Supplementary material for: Ion exchange chromatography as a simple and scalable method to isolate biologically active small extracellular vesicles from conditioned media
Source: PLoS One. 2023 Sep 15;18(9):e0291589. doi: 10.1371/journal.pone.0291589 (PMC10503763; doi:10.1371/journal.pone.0291589)
Supplement: S1 Table — Mean size, mode, D10, D50 and D90 parameters from the tRPS analysis for fractions 2, 3, 4 and 5. (DOCX) [file pone.0291589.s007.docx]

**Supplementary material**

**Supplementary Table 1**


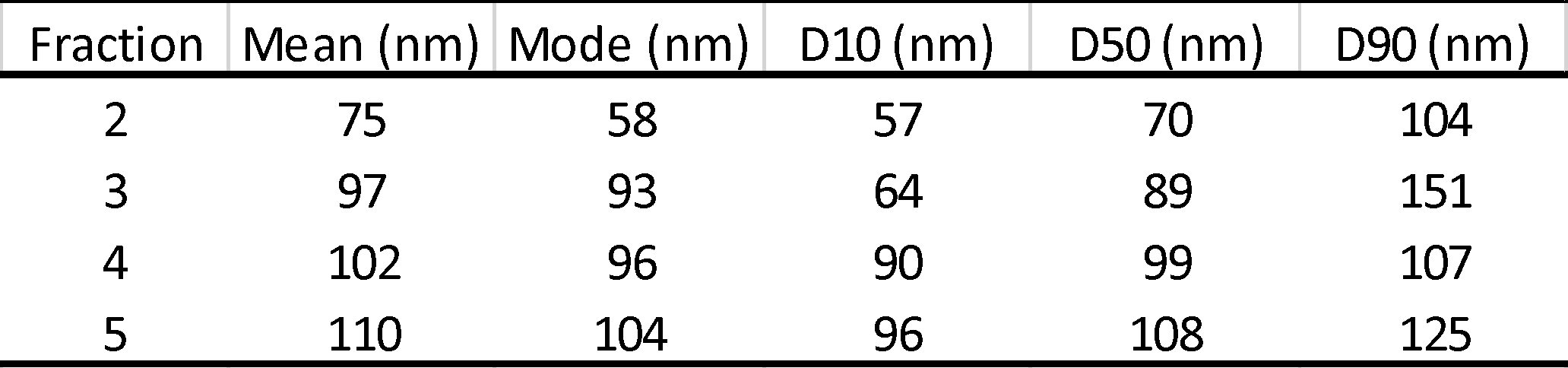


**Supplementary** **Table 1. Size and distribution parameters.** Mean size, mode, D10, D50 and D90 parameters from the tRPS analysis for fractions 2, 3, 4 and 5.
